# The bone rigidity error as a simple, quantitative, and interpretable metric for patient-specific validation of deformable image registration

**DOI:** 10.1016/j.phro.2025.100767

**Published:** 2025-04-23

**Authors:** Andreas Smolders, Tony Lomax, Francesca Albertini

**Affiliations:** a Centre for Proton Therapy, Paul Scherrer Institute, 5232 Villigen, Switzerland; b Department of Physics, ETH Zurich, 8092 Zurich, Switzerland

**Keywords:** Deformable image registration, Quality assurance, Adaptive radiotherapy, Physical plausibility

## Abstract

**Background and Purpose:**

Despite its potential, deformable image registration (DIR) is underutilized clinically, especially in time-sensitive cases, due to a lack of comprehensive metrics for assessing solution quality. Here, we propose a metric of physical plausibility, the bone rigidity error (BRE), that penalizes non-rigid transformations within individual bones, based on the assumption that bones do not deform.

**Materials and Methods:**

The BRE is calculated by segmenting bones individually and isolating the vectors of a deformable vector field within each bone. A rigid registration is least-square fitted to these vectors, and the BRE is calculated as the average deviation of these vectors from the fitted rigid registration. A lower BRE indicates better rigidity preservation. We evaluated the BRE for 6 DIR algorithms on 32 patients with 137 computed tomography (CT)-to-CT registrations across relevant anatomical sites.

**Results:**

The BRE varied widely between DIR algorithms, up to a factor of 3 on average for inhale-to-exhale thoracic CT registration. Despite large BRE differences between anatomical sites within each algorithm, some algorithms consistently outperformed others. Notably, a low BRE was not correlated with poorer image similarity, and the BRE was only weakly correlated to target registration error. Furthermore, we proposed bone-specific inspection thresholds for patient-specific validation. BRE calculation required less than 5.5 s.

**Conclusions:**

The BRE is an automatic, interpretable, fast, and easy-to-implement metric to assist validation of DIR algorithms, which show widely varying performance. It provides a useful complementary metric for patient-specific validation, especially in time-sensitive applications.

## Highlights

• This metric measures the non-rigid part of transformations in individual bones.
• Some deformable registration algorithms violate bone rigidity stronger than others.
• The bone rigidity error is reported in millimeters, facilitating interpretation.
• Evaluation of this metric is fast enough for online adaptive radiotherapy.

## 1 Introduction

Deformable image registration (DIR), the process of finding a deformable transformation that maps one image onto another one, has many applications in biomedical image processing [1]. In radiotherapy, DIR can be used for contour propagation, dose warping, and image fusion [2], [3]. Despite the promising treatment advances enabled by DIR, its clinical adoption, though increasing, remains slow due to reliability concerns.

This clinical hesitation is largely due to two reasons. First, because of the many degrees of freedom, the information in the registered images is generally insufficient to find a unique solution. Algorithms therefore impose additional constraints, like smoothness or invertibility, and because of the wide variety of options, they yield different solutions [3]. Second, effective quantitative metrics to assess the quality of a solution are lacking, especially on a patient-specific basis [4]. Several authors have proposed methods and recommendations to validate DIR solutions [5], [6], but the variety of DIR solutions combined with the difficulty in assessing their quality still hampers routine integration in clinical workflows.

Existing metrics to evaluate DIR are either operator-dependent, i.e. require manual annotations, or automatic [4], [6]. The most common operator-dependent metrics are the target registration error (TRE), based on manually annotated landmarks, or dice similarity (DSC) and mean distance to agreement (MDA), based on manual contours. Whilst powerful, they suffer from inter-operator variability, are sparse, and are highly time-intensive, making them impractical for routine patient-specific validation, especially in time-sensitive applications like adaptive radiotherapy [5], [7], [8].

Automatic metrics are either image-based or deformable vector field (DVF)-based. For image-based metrics, the reader is referred to [4]. DVF-based metrics evaluate the physical plausibility of a solution. The most common one is the Jacobian determinant [3], which >1 in case of expansion and <1 in contraction. Negative values or large local changes may indicate non-physical motion, and thus a DIR error [4]. The Jacobian determinant is fast and simple to calculate but may be hard to interpret. For example, sliding motion between organs may also yield large local changes, but they are physical. Another group of DVF metrics assess consistency [3], e.g. the inverse consistency, measuring whether the forward and backward registration are equal in magnitude and opposite in direction.

The main limitation of DVF-based metrics is that they may be fulfilled, although the deformation is incorrect. By evaluating multiple complementary metrics simultaneously, the plausibility of a wrong DIR solution passing all tests reduces. Another complementary metric could be based on the rigidity of individual bones. Although the transformation in DIR is deformable, within each individual bone it should in fact be rigid, although different bones may have different rigid transformations. Whereas some DIR algorithms directly enforce this [9], [10], [11], most algorithms do not, especially those used routinely.

Therefore, we aim to develop a fully automatic metric to assess the rigidity of bones in DIR. We propose the *bone rigidity error (BRE)*, penalizing deviations from the least squares fitted rigid transformation within each bone individually. The BRE is a distance, in millimeters, which facilitates interpretation by non-DIR experts. It is bone-specific, so it can trigger inspection of the DVF in a specific anatomical location. Its calculation is easy to implement and fast, making it applicable for online adaptive radiotherapy.

## 2 Materials and methods

### 2.1 Bone rigidity error

The BRE is calculated by first (automatically) segmenting each individual bone in the fixed image. A bone is defined here as a single connected piece of bone, e.g. the humerus or a single rib.

The BRE is then calculated by extracting the deformation $d_{i}$ for each coordinate $x_{i}$ of the DVF within a bone, with i the index of a discrete set of coordinates (e.g. for every voxel). This results in two sets of corresponding points, $x_{i}$ and $y_{i}=x_{i}+d_{i}$, for which the least-squares rigid registration can be found with

(1) $$\underset{R\in R^{3\times 3},t\in R^{3}}{\min}\;\overset{N}{\underset{i=1}{\sum }}{\left\|\left(Rx_{i}+t\right)-y_{i}\right\|}^{2}$$

$$\text{s.t.}\;RR^{T}=I$$

with R the rotation matrix, t the translation vector and N the number of voxels in the bone. This problem can efficiently be solved with the following steps [12]:

1. Calculate the centroids of each set of points $c_{X}=\frac{1}{N}\sum_{i=1}^{N}x_{i}$ and $c_{Y}=\frac{1}{N}\sum_{i=1}^{N}y_{i}$.
2. Defining $X=\left[x_{1},x_{2},\ldots ,x_{N}\right]$ and $Y=\left[y_{1},y_{2},\ldots ,y_{N}\right]$, calculate the 3 × 3 accumulation matrix $H=\left(X-c_{X}\right){\left(Y-c_{Y}\right)}^{T}$
3. Decompose H=UΣV with the singular value decomposition.
4. If $\left|VU^{T}\right|>0$, the optimal rotation $R^{*}=UV^{T}$. Else, $R^{*}=V^{\prime }U^{T}$, with $V^{\prime }$ the same matrix as V but with a change in sign in the third column.
5. The optimal translation can be found with $t^{*}=c_{Y}-Rc_{X}$

The BRE is then defined as the average distance between the least-squares rigid transformation and the deformed vectors

(2) $$BRE=\frac{1}{N}\overset{N}{\underset{i=1}{\sum }}\|\left(R^{*}x_{i}+t^{*}\right)-y_{i}\|.$$

The entire calculation of the BRE can be implemented in less than 20 lines of code in most modern programming languages, as shown for Python in the Supplementary Material.^1^

Importantly, the least-squares rigid transformation is not necessarily the ground truth: a DVF can have BRE=0 without being correct. Indeed, if e.g. the BRE of a global rigid registration is calculated, it will be 0, but this registration is not necessarily correct. A low BRE only means that the DVF maintains the rigidity of the bone, providing a physical plausibility to the result, and is thus a necessary condition rather than a sufficient one.

### 2.2 Implementation

We tested the BRE by calculating it for several different datasets and DIR algorithms. This allows to evaluate which algorithm best maintains the bone rigidity for several anatomical regions and clinical scenarios. We further evaluated the mean-absolute-error (MAE) between images and target registration error (TRE) between landmarks for comparison.

We used the open-source deep-learning segmentation tool Totalsegmentator [13] to segment 63 independent bone classes (Table 1 and Supplementary Table S1). All results were visually inspected and judged sufficient for this study. We calculated the BRE on the CT voxel level, and resampled the DVF using trilinear interpolation to find the deformations $d_{i}$. The metric was implemented in Python using Pytorch for GPU acceleration.

**Table 1:** Average bone rigidity error (BRE) [mm] for each dataset. The BRE was first averaged within each group (e.g. all ribs, left and right hip) and then averaged over the groups. Detailed results can be found in Supplementary Tables S2–S6.

Dataset	Plastimatch b-spline	Plastimatch demons	Velocity	Mirada	Raystation Anaconda	Cosylab DirOne
HNC	1.08	**0.84**	1.08	1.33	0.97	0.93
NSCLC	1.83	1.34	2.21	1.18	1.37	**0.82**
DIRLAB	0.85	0.48	1.15	0.86	0.56	**0.37**
APC	2.13	1.13	2.03	/	1.20	**1.10**
FULLBODY	1.23	**0.83**	1.55	/	0.99	0.85

### 2.3 Datasets

We used 5 datasets. The **HNC dataset** contained 5 patients with head-and-neck cancer, each having one planning CT and 4–7 repeated CTs, acquired throughout the treatment. Each repeated CT was registered to the planning CT, yielding 28 CT-CT pairs. The **NSCLC dataset** contained 5 patients with non-small-cell lung cancer (NSCLC), each having one planning CT and 9 repeated CTs, acquired in groups of 3 on 3 different days throughout the treatment [14], [15], [16], [17]. Each repeated CT was registered to the planning CT, yielding 45 CT-CT pairs. The public **DIRLAB dataset** consisted of 10 patients with the in- and exhale phase of 4D lung CT scans [18], [19]. Each exhale scan was registered to the corresponding inhale scan, yielding 10 CT-CT pairs. The **APC dataset** contained 5 patients, of which one pediatric, with various indications in the abdomen and pelvis regions. Each patient had a planning CT and 1–9 repeated CTs acquired throughout the treatment. Each repeated CT was registered to the planning CT, yielding 21 CT-CT pairs. The **FULLBODY dataset** contained 7 patients, of which five pediatric, with a planning CT and 2–7 repeated CTs acquired through the treatment. All CT scans covered the entire body except the legs. Each repeated CT was registered to the planning CT, yielding 33 CT-CT pairs.

### 2.4 DIR algorithms

We used the BRE to evaluate 6 DIR algorithms: plastimatch b-spline and demons [20], Varian Velocity (Varian Medical Systems, Palo Alto, USA), Mirada CT Deformable (Mirada Medical, Oxford, UK), Raystation Anaconda (RaySearch Laboratories AB, Stockholm, Sweden) and Cosylab DirOne (Cosylab, d. d. Control System Laboratory, Ljubljana, Slovenia). Details on these algorithms and their hyperparameters can be found in the Supplementary Material.

**Fig. 1:**
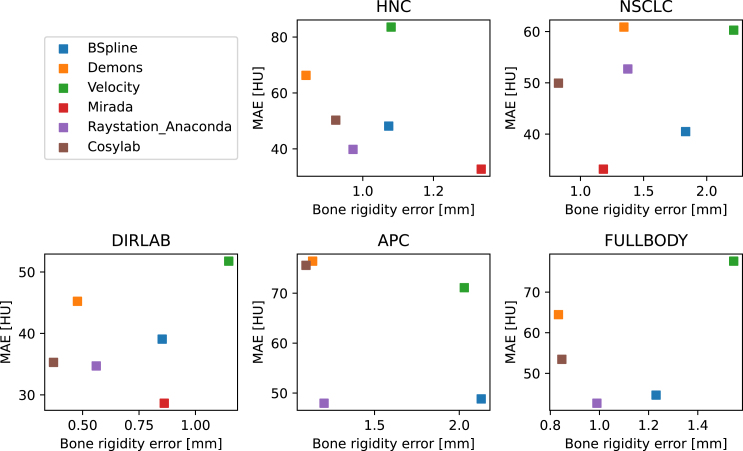
Average bone rigidity error (BRE) versus average mean absolute error (MAE) for the different datasets and DIR algorithms.

**Fig. 2:**
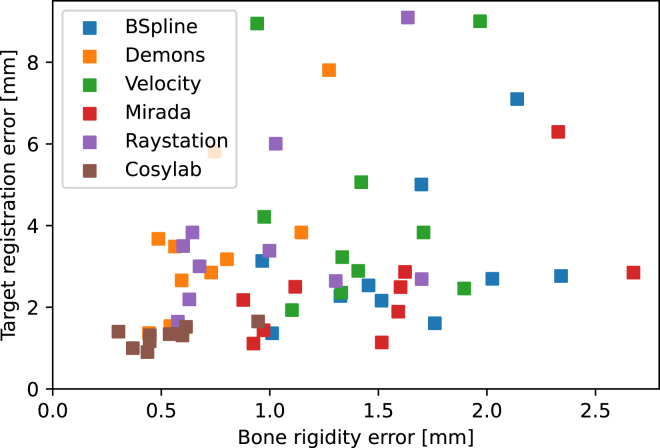
Bone rigidity error (BRE) versus target registration error (TRE) for the DIRLAB scans for all DIR algorithms. Each registration of each patient with each DIR is plotted as a single point in the scatter plot. Note that the landmarks in the DIRLAB dataset are inside the lung and not inside the bone, partly obscuring the relation between the BRE and TRE.

**Fig. 3:**
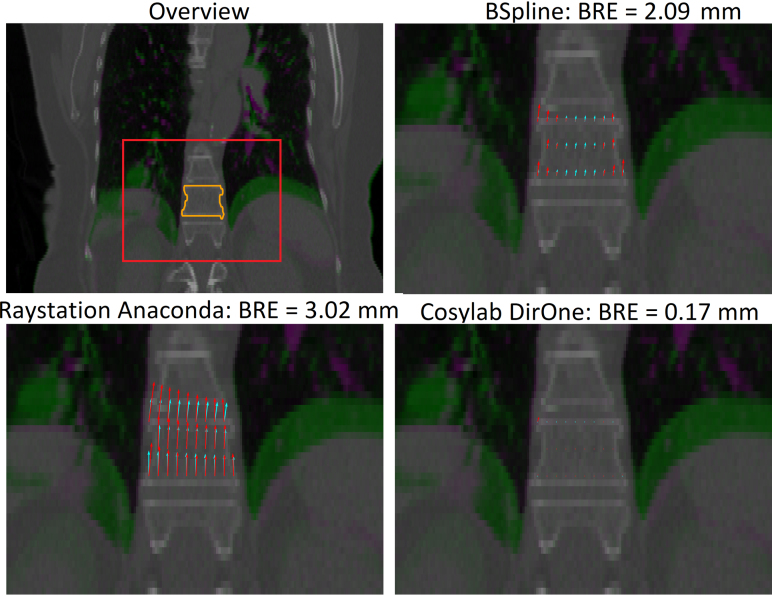
Visualization of the BRE in vertebra T11 of DIRLAB patient 10. The DVF of plastimatch b-spline, Raystation Anaconda, and Cosylab DirOne are indicated in red arrows, together with their corresponding least-squares rigid vector field in cyan. Note that the fitted rigid vector fields are not the same for the three DIR algorithms, as they represent the least-squares rigid transformation of each DVF, and not a single ground truth. (For interpretation of the references to color in this figure legend, the reader is referred to the web version of this article.)

**Fig. 4:**
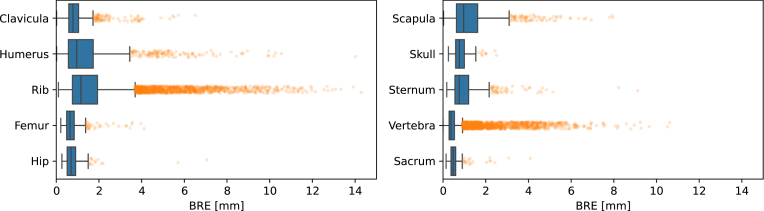
Boxplots of the BRE in different groups of bones. Each box contains points for each of the 137 scans, each DIR algorithm (except Velocity, because of the poor performance on both the MAE and BRE), and each individual bone (e.g. multiple vertebra or ribs). (For interpretation of the references to color in this figure legend, the reader is referred to the web version of this article.)

**Fig. 5:**
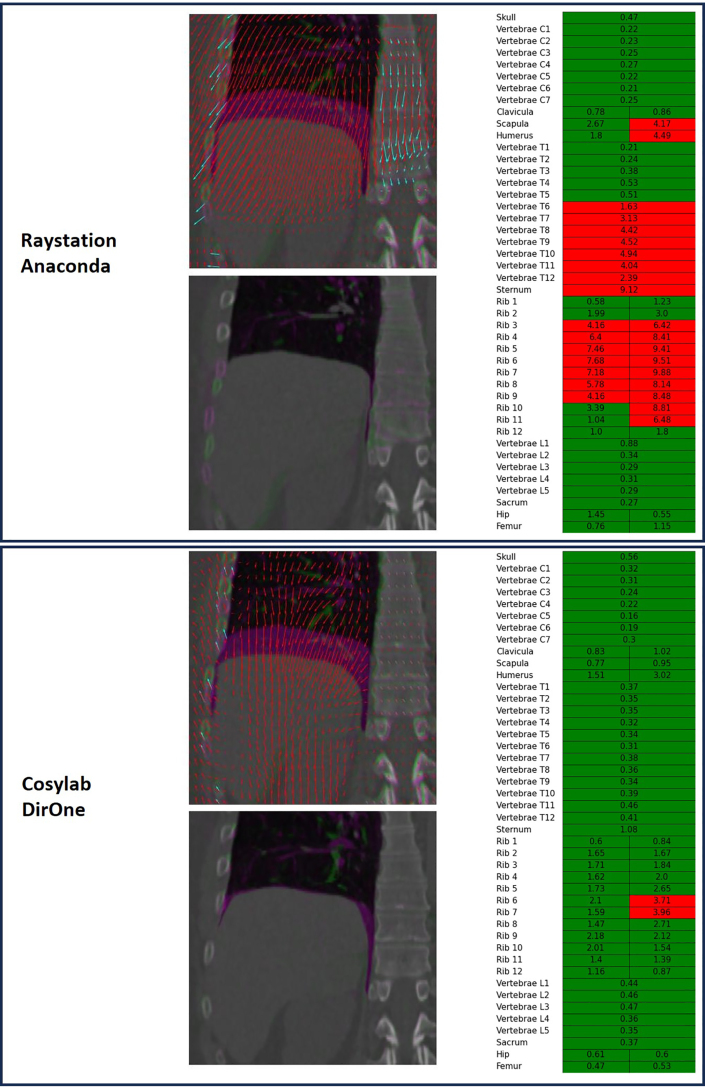
Visualization of a patient-specific validation for a patient in the FULLBODY dataset. The registration with Raystation Anaconda and Cosylab DirOne are shown. The left figures show an overlay of the fixed (purple) and moving (green) images, together with the DVF in red. The cyan vectors are the corresponding least-squares rigid vector fields for each of the bones. The middle figures show an overlay of the fixed (purple) and moved (green) images. On the right, the BRE for all bones is given, in red if the threshold is surpassed and in green if not. (For interpretation of the references to color in this figure legend, the reader is referred to the web version of this article.)

## 3 Results

The BRE varied largely across the DIR algorithms (Table 1, with bone-specific results in Supplementary Tables S2–S6). Overall, Cosylab DirOne appeared to preserve bone rigidity most effectively, closely followed by Plastimatch demons. In contrast, Velocity performed the worst, while the Plastimatch b-spline also notably violated the rigidity of individual bones. The disparity in BRE between the best and worst-performing algorithms reached up to a factor 3 for the DIRLAB dataset, underscoring the importance of validating BRE on an algorithm-specific basis.

Substantial differences were also observed between the datasets, i.e. between anatomical regions (Table 1). Moreover, the difference between the NSCLC and DIRLAB datasets was also large, although both involve the thorax. The DIRLAB dataset included phases of a 4D CT, while the NSCLC dataset captured interfractional changes, indicating that the type of deformation also affects the BRE. Although the absolute BRE values varied considerably between datasets, the relative performance of the algorithms remained consistent, e.g., an algorithm performing well relative to others on the HNC dataset also demonstrated strong performance on the FULLBODY dataset.

In many DIR algorithms, the optimization involves balancing image similarity with DVF smoothness or physical plausibility. The BRE serves as a metric for physical plausibility, so one could expect that algorithms with a low BRE would result in poorer image similarity. However, this was not observed (Fig. 1). When the average MAE of the transformed images was compared against the BRE across different algorithms and datasets, no clear correlation was found. Some algorithms exhibited both high MAE and BRE (e.g., Velocity), while others, for certain datasets, achieved both low BRE and MAE simultaneously (e.g., Raystation Anaconda on the APC datasets).

The gold standard for assessing registration quality is the TRE, which, however, requires landmarks. Ideally, BRE would correlate with TRE, allowing it to serve as a substitute metric for evaluating DIR quality without landmarks. For the DIRLAB dataset, such landmarks were available. Although these landmarks were inside the lung and not in the bone, the TRE and BRE were still partly linked through the smoothness of the DVF. Comparing the TRE to the BRE yielded a Pearson correlation coefficient of 0.37, indicating only a modest positive correlation (Fig. 2). Thus, BRE was linked to TRE, but cannot substitute for it.

The BRE notably differed between DIR algorithms in the vertebrae of the DIRLAB dataset. Cosylab DirOne achieved an average BRE of 0.09 mm, 5 to 10 times lower than that of any other algorithm. This discrepancy was due to the sliding boundary of the lung. The upward movement of the lung, combined with the smoothness constraints imposed by DIR algorithms, caused deformation of the vertebrae, which should have remained rigid (Fig. 3). Remarkably, Cosylab DirOne avoided this issue while still maintaining a low TRE (Fig. 2). An example of how this translates into dosimetric errors after dose warping is given in Supplementary Figure S1.

The largest variations in BRE were however not between algorithms or datasets, but between different bones (Fig. 4 and Supplementary Tables S2–S6). For thin, elongated bones such as the ribs, humerus, or scapula, the BRE often reached up to 4 mm. In contrast, for shorter, more rounded bones like the vertebrae or sacrum, the BRE generally remained below 1 mm. This suggests that, when assessing DIR quality based on BRE, different bones should be evaluated using different criteria. For patient-specific validation, it is beneficial to establish thresholds for flagging cases or regions where results cannot be trusted or require further inspection. Although such thresholds are inherently arbitrary, we propose using a limit for outliers analogous to that used in boxplots; that is, the orange points in Fig. 4 indicate cases requiring further scrutiny. The suggested bone-specific thresholds are provided in Supplementary Table S7.

Using the proposed thresholds, the bones where the BRE exceeds the acceptable limits can be highlighted to guide the inspection (Fig. 5). Given that difficulties or errors in the registration process often impact multiple bones simultaneously, several bones are typically flagged together (Fig. 5).

The time required to calculate the BRE varied depending on the number of bones involved in each dataset (Supplementary Table S8), but even for the FULLBODY dataset, including CT scans from the head to the pelvis, the total time was only 5.41 s, showing that the BRE can be calculated fast enough for online applications. This time did not include the automatic segmentation, which took around 1 min.

## 4 Discussion

In this work, we proposed the *bone rigidity error (BRE)*, a metric for patient-specific DIR validation that penalizes the non-rigid transformations of individual bones. We demonstrated that the BRE was quick to calculate, simple to implement, and that different algorithms performed very differently with respect to it.

Because it does not require manual annotations, its speed, and its interpretability, the BRE is particularly advantageous for time-sensitive applications like adaptive therapy [5], [8]. The BRE is most beneficial if global DIR quality is required, i.e. the quality everywhere is important and not just around the tumor. Examples of such applications are dose warping or image fusion where the fused image is used for dose calculation [3], [21]. Although it may also be useful for structure propagation, direct inspection of the propagated structures is likely still easier [6]. For global DIR quality assessment, the BRE adds value over the Jacobian determinant [3] because it can assess the rigidity of the entire bone, rather than just volume changes. While rigidity implies incompressibility, the reverse is not true. In practice, this means that even if the Jacobian determinant averaged over a bone is close to 1, the bone may still undergo non-rigid transformations, such as shear, as also shown in Supplementary Figure S1. The BRE is therefore more stringent while still necessary. The Jacobian determinant is still useful for soft tissue, where volume expansions and contractions are also relevant.

DIR algorithms often involve trade-offs, e.g. image similarity versus DVF smoothness or high quality in one region versus another. Most applications in radiation oncology would not compromise other metrics to improve DIR plausibility in the bone, and hence would not want to improve BRE if it compromises something else. However, from all our analysis with other DIR metrics, we found that a good BRE did not seem to compromise other quality metrics, and is therefore simply an improvement of the global DIR quality. These findings were consistent with additional analyses performed on standard clinically used metrics, including Mean Surface Distance, Surface Dice, and Dice, for a large set of structures for HNC and NSCLC datasets, which showed no evidence that achieving a good BRE compromises the quality in soft tissue (data not reported).

To further facilitate patient-specific validations, we proposed bone-specific inspection thresholds based on our observed data. While this is practically useful, such thresholds should ideally be based on some physical metric, e.g. expected deformations or mechanical strains in the bones. However, due to the highly complex and varying shapes of the bones, establishing general thresholds based on physical quantities is highly complicated. We hypothesize that biomechanical models based on an average bone shape could help in establishing more accurate thresholds, but this goes beyond the scope of the current article.

The BRE varied widely between algorithms. Interestingly, Plastimatch b-spline, Velocity and Cosylab DirOne all use a b-spline deformation model, but still yield different BRE, with Velocity even the worst of all and Cosylab DirOne the best. This could in part be based on the grid spacing, but this could not be verified as the grid spacing of Velocity and Cosylab DirOne was not reported. A complete study of which DIR algorithm parameters yield good BRE values lies beyond the scope of this article, but is an interesting direction for future work.

Like most DVF-based metrics, the BRE is a necessary but not sufficient condition for assessing DIR quality [4]. For instance, bad registrations, such as global rigid or highly smooth ones although deformation occurs, could still yield a low BRE. Therefore, the BRE should be considered an additional metric, used complementarily with others, including image-similarity, DVF, and observer-dependent metrics. Furthermore, the BRE exclusively assesses physical plausibility within the bones and may overlook issues occurring outside the bones, particularly when those issues are distant from the evaluated bones.

Importantly, the BRE should not be used for a bone that can transform non-rigidly. For example, for reirradiation of a pediatric patient in adulthood, the bones will have grown, and the assumption that bones transform rigidly does not hold. Other examples of violations of this assumption are bone fractures, surgical removal of a part of a bone or bone tumors.

The BRE depends on the segmentation of individual bones. With advances in deep-learning segmentation, accurate automatic segmentation has become feasible [22], particularly bones on CT scans. In this work, we utilized an open-source tool that can be easily downloaded by anyone for validating their DIR algorithms. For online adaptation, the time required for auto-contouring may be a concern [5], although the tool we used took only about 1 min. Since auto-contouring can be performed concurrently with the DIR algorithm, we anticipate minimal delays in the overall process. Furthermore, if the daily acquired scan is used as the moving image, auto-contouring can be completed before its acquisition, as evaluating the BRE only requires the fixed image needs to be segmented and potentially inspected for erroneous bone segmentation.

Our work focused on CT-to-CT registration. However, the BRE is likely even more relevant in multimodal registration, such as CT to MRI or CT to PET, which are generally more challenging [23]. For image fusion in radiotherapy, where the fixed image is a CT scan allowing for straightforward bone segmentation, the BRE remains as easy to evaluate as in our current case, whereas other metrics may become more challenging to apply.

Ideally, the BRE could be incorporated as a regularization term in the optimization function of the DIR algorithm itself, similar to [24], which could achieve a similar effect as enforcing the rigidity of bones as in some biomechanical DIRs [9], [10]. Although we did not have access to a high-quality DIR algorithm in which to integrate this metric, we believe that such an extension would be straightforward to implement with minimal impact on runtime, particularly if the segmentation is performed in advance. Whereas a similar objective could be achieved with a hybrid DIR with bones as guiding structures, the advantage of the BRE is that it only requires the segmentations in one scan, instead of both.

In conclusion, the bone rigidity error is a fast and easy-to-implement metric to validate the physical plausibility of DIR algorithms. We showed that the BRE widely varies between DIR algorithms, showing that it can be used to compare various algorithms. For patient-specific validation, it provides a useful complementary metric.

## CRediT authorship contribution statement

**Andreas Smolders:** Conceptualization, Methodology, Software, Investigation, Formal analysis, Writing – original draft, Data curation, Visualization. **Tony Lomax:** Conceptualization, Writing – review & editing, Funding acquisition, Resources, Supervision. **Francesca Albertini:** Conceptualization, Writing – review & editing, Funding acquisition, Project administration, Resources, Supervision.

## Declaration of competing interest

The authors declare that they have no known competing financial interests or personal relationships that could have appeared to influence the work reported in this paper.

Francesca Albertini is a Guest Editor for this journal and was not involved in the editorial review or the decision to publish this article.

## Appendix A Supplementary data

MMC S1 .

## Footnotes

1 The code is further available at https://github.com/AndreasSmolders/BoneRigidityError.
