# Supplementary material for: The bone rigidity error as a simple, quantitative, and interpretable metric for patient-specific validation of deformable image registration
Source: Phys Imaging Radiat Oncol. 2025 Apr 23;34:100767. doi: 10.1016/j.phro.2025.100767 (PMC12127556; doi:10.1016/j.phro.2025.100767)
Supplement: MMC S1 — . [file mmc1.pdf]

Supplementary material

DIR algorithm details

DIR algorithms often employ a b-spline transformation model because of its speed and good results [1, 2]. We therefore included the `plastimatch` b-spline algorithm [3], using the mean-squared-error (MSE) as similarity criterion, two consecutive resolutions with grid spacing 40 and 20 mm and regularization $\lambda_r = 0.002$. These parameters are provided as examples in the `plastimatch` documentation and were also used in [4, 5]. An initial automatic rigid alignment was also included. Furthermore, we included the `plastimatch` demons algorithm. Demons is a commonly used parametric DIR algorithm. [6]. The implementation details followed the default from the documentation and can be found in [4]. The algorithm also included an initial automatic rigid alignment.

Next to these two open-source implementations, we included 4 commercial DIR algorithms. Unfortunately, the exact implementation details of these algorithms are partially proprietary, and we can only report what is public. For all algorithms, the default parameters provided by the vendors were used, which reflects usual clinical practice. The algorithms included the *extended deformable multi pass* in Velocity (Varian Medical Systems, Palo Alto, USA). This b-spline-based DIR algorithm uses 6 consecutive grid spacings with initial rigid alignment, but the resolutions are not reported to the user. Also the *CT Deformable* algorithm by Mirada (Mirada Medical, Oxford, UK) was included. This algorithm is based on optical flow, i.e. a parametric DIR algorithm like Demons, but using diffusion partial differential equations instead of Gaussian smoothing [7]. The Mirada algorithm was not evaluated on the FULLBODY and APC datasets due to the loss of access during the evaluation period. Further, we included the intensity-based ANACONDA algorithm in Raystation (RaySearch Laboratories AB, Stockholm, Sweden) [8] without guiding structures, with 3 consecutive resolutions from 5 to 2.5 mm. ANACONDA also employs a regularization term to ensure smoothness. Finally, we included a preclinical DIR algorithm DirOne provided by Cosylab (DirOne, Cosylab, d. d. Control System Laboratory, Ljubljana, Slovenia) [9]. DirOne also uses a b-spline transformation model, but uses discrete optimization with a minimum spanning tree, like the *deeds* algorithm [10].

BRE python code

The following function defines a `Python` implementation of the bone rigidity error. It uses pytorch for GPU acceleration, but the keyword `torch` could be integrally changed with `numpy`.

```python
import torch
def BRE_torch(X, Y):
    assert X.shape == Y.shape
    assert X.shape[0] == Y.shape[0] == 3
    centroid_X = torch.mean(X, axis=1)
    centroid_Y = torch.mean(Y, axis=1)
    Xm = X - centroid_X
    Ym = Y - centroid_Y

    H = Xm @ torch.transpose(Ym,0,1)
    U, S, Vt = torch.linalg.svd(H)
    R = Vt.T @ U.T

    if torch.linalg.det(R) < 0:
        Vt[2,:] *= -1
        R = Vt.T @ U.T

    t = -R @ centroid_X + centroid_Y
    BRE = torch.mean(torch.linalg.norm(Y-(R@X+t), axis=0)))
    return BRE
```

Additonal figures and tables

Table S1: Summary of the 63 bone classes used in this work

skull	vertebrae T8	rib right 8
vertebrae C1	vertebrae T9	rib left 9
vertebrae C2	vertebrae T10	rib right 9
vertebrae C3	vertebrae T11	rib left 10
vertebrae C4	vertebrae T12	rib right 10
vertebrae C5	sternum	rib left 11
vertebrae C6	rib left 1	rib right 11
vertebrae C7	rib right 1	rib left 12
clavicula left	rib left 2	rib right 12
clavicula right	rib right 2	vertebrae L1
scapula left	rib left 3	vertebrae L2
scapula right	rib right 3	vertebrae L3
humerus left	rib left 4	vertebrae L4
humerus right	rib right 4	vertebrae L5
vertebrae T1	rib left 5	sacrum
vertebrae T2	rib right 5	hip left
vertebrae T3	rib left 6	hip right
vertebrae T4	rib right 6	femur left
vertebrae T5	rib left 7	femur right

Table S2: Bone rigidity error [mm] for the HNC dataset.

Bone	*Nbones*	Plastimatch b-spline	Plastimatch demons	Velocity	Mirada	Raystation Anaconda	Cosylab DirOne
Clavicula	56	1.07	0.80	1.05	1.23	0.83	0.90
Humerus	56	1.14	0.86	1.17	1.45	1.42	0.91
Rib	286	1.55	0.90	1.04	2.07	0.95	1.44
Scapula	56	1.10	1.49	2.05	1.53	1.89	1.02
Skull	28	1.02	0.85	0.84	1.29	0.53	0.67
Sternum	28	1.16	0.56	0.81	1.30	0.81	1.14
Vertebra	337	0.49	0.41	0.61	0.47	0.38	0.40
Mean		1.08	**0.84**	1.08	1.33	0.97	0.93

Table S3: Bone rigidity error [mm] for the NSCLC dataset.

Bone	*Nbones*	Plastimatch b-spline	Plastimatch demons	Velocity	Mirada	Raystation Anaconda	Cosylab DirOne
Clavicula	90	1.41	1.02	2.12	1.09	1.28	0.76
Humerus	90	2.28	1.61	2.34	1.49	1.70	1.03
Rib	1080	2.89	1.62	2.90	1.93	1.48	1.24
Scapula	90	1.88	1.97	2.38	1.08	1.72	0.93
Sternum	45	0.96	0.83	1.87	0.74	1.31	0.48
Vertebra	765	1.57	1.02	1.67	0.76	0.75	0.51
Mean		1.83	1.34	2.21	1.18	1.37	**0.82**

Table S4: Bone rigidity error [mm] for the DIRLAB dataset.

Bone	$Nbones$	Plastimatch b-spline	Plastimatch demons	Velocity	Mirada	Raystation Anaconda	Cosylab DirOne
Clavicula	20	0.52	0.28	0.99	0.53	0.33	0.36
Humerus	18	0.14	0.19	0.93	0.31	0.14	0.29
Rib	226	2.56	1.03	1.78	2.39	1.47	0.88
Scapula	20	0.43	0.29	1.08	0.50	0.30	0.16
Sternum	10	0.75	0.58	1.09	0.76	0.55	0.46
Vertebra	137	0.71	0.49	1.01	0.68	0.57	0.09
Mean		0.85	0.48	1.15	0.86	0.56	**0.37**

Table S5: Bone rigidity error [mm] for the APC dataset.

Bone	$Nbones$	Plastimatch b-spline	Plastimatch demons	Velocity	Mirada	Raystation Anaconda	Cosylab DirOne
Femur	40	1.16	0.94	1.44	/	0.73	0.74
Hip	42	0.92	0.98	1.49	/	0.57	0.77
Humerus	29	2.68	2.04	2.88	/	1.83	1.70
Rib	175	5.20	1.52	3.68	/	2.95	1.99
Sacrum	21	0.76	0.50	1.29	/	0.30	0.56
Sternum	10	3.57	1.31	2.34	/	1.54	1.38
Vertebra	131	0.63	0.66	1.09	/	0.49	0.52
Mean		2.13	1.13	2.03	/	1.20	**1.10**

Table S6: Bone rigidity error [mm] for the FULLBODY dataset.

Bone	$Nbones$	Plastimatch b-spline	Plastimatch demons	Velocity	Mirada	Raystation Anaconda	Cosylab DirOne
Clavicula	66	0.88	0.64	1.56	/	0.76	0.88
Femur	66	0.74	0.56	1.33	/	0.82	0.67
Hip	66	0.86	0.80	1.50	/	0.80	0.59
Humerus	66	2.55	1.70	2.18	/	1.94	1.77
Rib	778	1.90	0.88	1.73	/	1.29	1.18
Sacrum	33	0.76	0.53	1.38	/	0.65	0.45
Scapula	66	1.26	1.20	1.84	/	1.52	0.82
Skull	33	0.90	0.91	1.76	/	0.58	0.72
Sternum	33	1.97	0.72	1.34	/	1.17	1.03
Vertebra	792	0.49	0.38	0.85	/	0.37	0.35
Mean		1.23	**0.83**	1.55	/	0.99	0.85

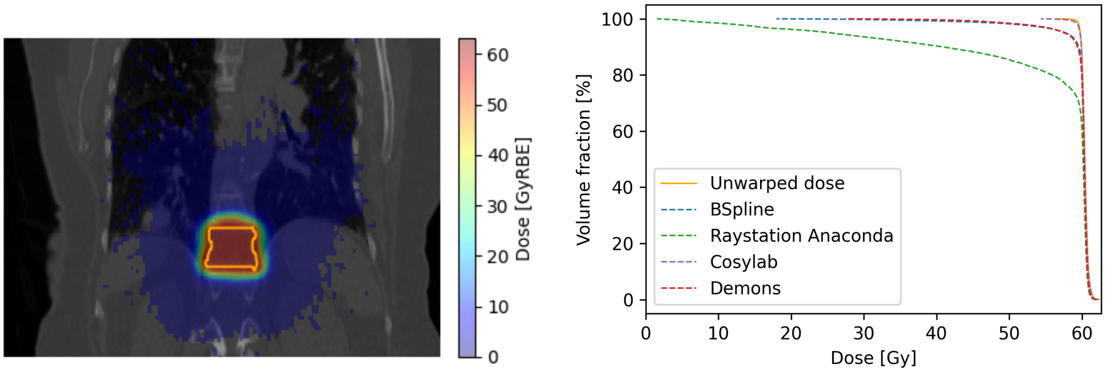

Algorithm	BRE [mm]	Jacobian determinant [-]
Plastimatch b-spline	2.09	0.96
Plastimatch Demons	1.14	1.02
Raystation Anaconda	3.02	1.12
Cosylab DirOne	0.17	1.00

Figure S1: Example of a case in which the BRE is useful to detect errors in dose warping. For the DIRLAB patient 10, also shown in Figure **??** of the manuscript, we designed a simple proton therapy treatment plan delivering a homogeneous 60 Gy dose to the vertebra T11 with two posterior fields on the *moving* scan (left plot). This results in a sharp DVH of this vertebra (Unwarped dose, right plot). This dose was warped to the fixed scan with several DIR algorithms, and the DVH of the warped doses are also plotted (right plot). The corresponding BRE and average Jacobian determinants for the T11 vertebra are given in the table. Only Cosylab DirOne retains the correct DVH after warping, related to a low BRE. Note that both Plastimatch b-spline and demons have Jacobian determinants close to 1, but do not result in accurately warped dose distributions. Even though this patient did not have a tumor in that vertebra, patients with chordomas or chondrosarcomas including irradiation of several vertebra are regularly treated with proton therapy, and such a dose distribution is therefore a realistic clinical example.

Table S7: Proposed thresholds for manual inspection of a DVF for each of the bones. The thresholds t were defined as $t = q3 + 1.5 * (q3 - q1)$, with $q1$ and $q3$ the first and third quartile of BRE values across all our datasets, inspired by the definition of the upper whisker in a boxplot. Because of its poor performance, the results of Velocity were excluded when defining these thresholds.

Bone	BRE threshold [mm]
Clavicula	1.74
Femur	1.38
Hip	1.50
Humerus	3.48
Rib	3.70
Sacrum	0.92
Scapula	3.11
Skull	1.61
Sternum	2.16
Vertebra	0.91

Table S8: Average time required to calculate the bone rigidity error. Time measurements were performed on a linux-based system with 8 Intel Xeon E3-1240 v5 CPU cores and an Nvidia Quadro P6000 GPU.

Dataset	Time [s]
HNC	1.71
NSCLC	2.32
DIRLAB	0.78
APC	1.36
FULLBODY	5.41

References

1. Rueckert D, Sonoda LI, Hayes C, Hill DLG, Leach MO, and Hawkes DJ. Nonrigid registration using free-form deformations: application to breast MR images. IEEE Trans Med Imaging 1999; 18:712–21. https://doi.org/10.1109/42.796284
2. Sotiras A, Davatzikos C, and Paragios N. Deformable Medical Image Registration: A Survey. IEEE Trans Med Imaging 2013; 32:1153–90. https://doi.org/10.1109/TMI.2013.2265603
3. Sharp GC, Li R, Wolfgang J, Chen G, Peroni M, Spadea MF, Mori S, Zhang J, Shackleford J, and Kandasamy N. Plastimatch: an open source software suite for radiotherapy image processing. *Proceedings of the XVI'th International Conference on the use of Computers in Radiotherapy (ICCR), Amsterdam, Netherlands.* 2010
4. Nenoff L et al. Deformable image registration uncertainty for inter-fractional dose accumulation of lung cancer proton therapy. Radiother Oncol 2020; 147:178–85. https://doi.org/10.1016/J.RADONC.2020.04.046
5. Smolders A, Lomax A, Weber D, and Albertini F. Patient-specific neural networks for contour propagation in online adaptive radiotherapy. Phys Med Biol 2023; 68:095010. https://doi.org/10.1088/1361-6560/accaca
6. Thirion JP. Image matching as a diffusion process: an analogy with Maxwell's demons. Med Image Anal 1998; 2:243–60. https://doi.org/10.1016/S1361-8415(98)80022-4
7. Lucas BD. Generalized image matching by the method of differences. Carnegie Mellon University, 1985
8. Weistrand O and Svensson S. The ANACONDA algorithm for deformable image registration in radiotherapy. Med Phys 2015; 42:40–53. https://doi.org/10.1118/1.4894702
9. Alshammari SS, Yaddanapudi S, Kušnik B, Ivančič R, Anderle K, Li JG, Furutani KM, Beltran CJ, and Lu B. Benchmarking and performance evaluation of a novel deformable image registration software for radiotherapy CT images. Tech Innov Patient Support Radiat Oncol 2024; 32:100295. https://doi.org/10.1016/j.tipsro.2024.100295
10. Heinrich MP, Jenkinson M, Brady SM, and Schnabel JA. Globally optimal deformable registration on a minimum spanning tree using dense displacement sampling. *Medical Image Computing and Computer-Assisted Intervention–MICCAI 2012: 15th International Conference, Nice, France, October 1-5, 2012, Proceedings, Part III 15.* Springer, 2012 :115–22
